# Epilepsy from Disease of Internal Ear

**Published:** 1877-03

**Authors:** R. W. Westmoreland

**Affiliations:** Atlanta, Ga.


### EPILEPSY FROM DISEASE OF INTERNAL EAR.

By R. W. WESTMORELAND, M.D., Atlanta, Ga.

Mr. ——, æt. 28, has been subject to epileptic attacks for ten or twelve years. One year before his first attack he had a spell of scarlet fever, which resulted in a chronic inflammation of the internal ear (right ear). The convulsions for the last eight or ten years have been occurring very frequently, averaging one every week, being ushered in without any premonitory symptoms, and causing complete temporary paralysis. In falling there is a tendency to turn, forwards, to the left. The eyes roll upward and are fixed, while he foams at the mouth. The breathing is labored, while the skin is congested and covered with perspiration. This extreme condition lasts about ten minutes, and then the symptoms begin to modify, and he returns to consciousness usually in fifteen to twenty, with no knowledge of what has occurred, and with no other immediate result than a slight headache. Then his bowels become costive, which before are regular, and the ear discharges an increased amount of pus.

The history of this patient did not indicate any hereditary predisposition, and while he had been treated variously for gastric disturbance, masturbation, etc., I could see nothing calculated to induce such a view of its etiology. I attributed it altogether to the cerebral irritation arising from the inflamed internal ear, and shaped my course accordingly.

The ordinary treatment for chronic inflammation of the ear was pursued, in connection with ammoniated copper, which had the effect of lengthening the periods between the attacks. After awhile the copper was changed for potass. bromide, the ear treatment being kept up, with marked benefit. Under this plan the patient went four or five months without a convulsion. At the end of this time he fell "in a fit," and hitting his shoulder against some projecting point, received a posterior dislocation of the humerus. In attempting to diagnose the injury, the pain consequent upon manipulation brought on a convulsion. He was given, hypodermically, an eighth of a grain of morphine, and again manipulation commenced, which, as before, excited convulsions. Upon this, ether was administered; and, with the assistance of Dr. W. F. Westmoreland, we reduced the dislocation. No further trouble was experienced, and the patient went some time without an attack, and when it did come it was noticed and remarked to be the lightest he had ever had.

Before or about this time, he was troubled with a general eruption on the skin, and, supposing that it was from the large doses of bromide potass. he was taking (gr. xx. *ter die*), I substituted the liquor bromidi arsenici, partially, for the bromide solution—that is, I gave the bromide morning and evening, and the liquor at noon. The eruption soon disappeared, and has never recurred. In connection with this last change, the patient has been restricted to animal diet, and is looking healthy and robust.

The ear is not yet well, but has improved considerably under the constant application of caustics and astringent lotions, in conjunction with the air douche, administered occasionally. I should also state, probably, that a seaton has been worn in the back of the neck for the last two or three months.

Now, the question arises, is this malady curable? Some say not; though it may disappear temporarily, it will return.

Especially is this the case, it is said, when the disease is hereditary, or is of such long standing that the nervous system has become too thoroughly impressed to recover entirely. Very often, when the exciting cause can be discovered and removed, and the predisposition of, or impression on, the nervous system is not too perfectly established, the disease may be cured. On the contrary, where these unfavorable conditions do certainly exist, the history of such cases compels us to infer that temporary amelioration is the best we may expect.

I feel no doubt that if the inflamed ear heals, this patient will entirely recover. I am led to this expectation by the fact that, even in the presence of the irritation, the remedies have not only controlled the difficulty, by lengthening the intermission of the "fits," but they are much less in severity, when they do come on.

In a notice of the treatment by the liquor bromidi arsen., Dr. Th. Clemens, of Frankfort-on-the-Main, speaks of astonishing results and benefit from the use of the preparation. This article is in the November number of *Druggists' Circular* of 1876, No. 11, page 186, where the preparation, dose, and advantages over the simple solution of bromide potass. are given.

The philosophy of its benefit is very reasonable, and I propose to keep this patient on it exclusively, as soon as he can get a tolerance of the preparation. On giving it at first it seemed to engender some difficulties in the stomach, but as soon as I lessened the dose to once instead of three times a day, no trouble was experienced.

